# Total organic carbon quantification in soils and sediments: Performance test of a modified sample preparation method

**DOI:** 10.1016/j.mex.2024.102934

**Published:** 2024-08-30

**Authors:** Moritz Nykamp, Fabian Becker, Philipp Hoelzmann

**Affiliations:** Freie Universität Berlin, Institute of Geographical Sciences, Malteserstr. 74–100, 12249 Berlin, Germany

**Keywords:** Direct TOC determination, Suspended particulate samples, Preparation of sample suspensions, Method validation, Certified reference materials, A modified preparation method for TOC determination in suspended particulate samples

## Abstract

The total organic carbon (TOC) concentration of particulate samples is a key parameter to characterize soils and sediments. To demonstrate the applicability and reliability of a modified sample preparation method for the direct measurement of TOC contents in suspended particulate samples, we analyzed five certified reference materials (CRMs) with varying TOC concentrations using a Shimadzu TOC-L CPH analyzer. Measured values were calibrated with a multi-point curve that cover the full range of the expected TOC concentrations and the results were validated using statistical values and measures. The method validation reveals that the measurements are accurate and precise for CRMs from marine and soil contexts, but show a low accuracy for the CRM containing polycyclic aromatic hydrocarbons (PAHs). This demonstrates the applicability and reliability of the modified preparation method for direct TOC determination of suspended particulate samples. Therefore, it is relevant for a broader community, beyond geosciences, and for users employing devices of other manufacturers to analyze TOC in suspended particulate samples.

• Modified preparation method uses reduced sample weights and yields accurate and precise results.
• Cost-efficient and environmentally friendly alternative: reduces waste by saving acid and ultrapure water.
• Avoids incomplete dissolution of dolomite by heating acidified samples.

## Graphical abstract

**Figure:**
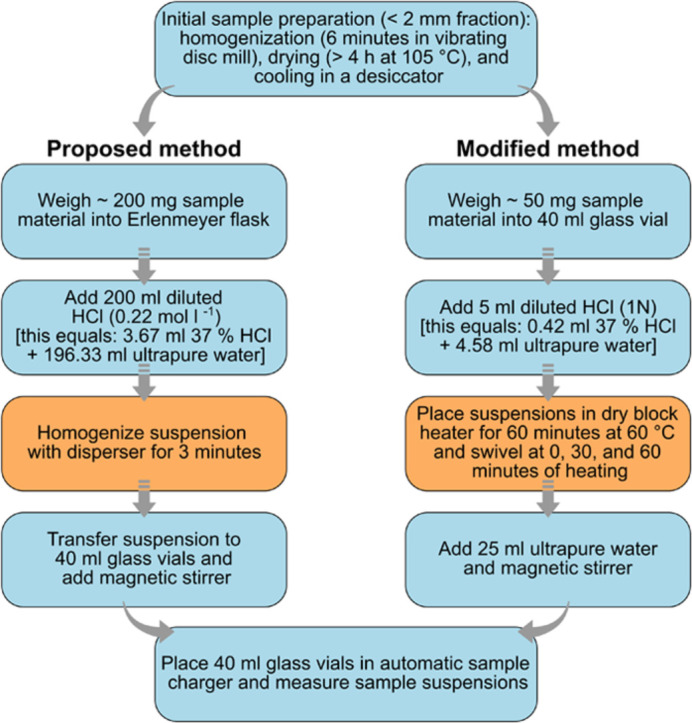

Specifications table

**Table:** 

Subject area:	Earth and Planetary Sciences
More specific subject area:	TOC determination in soils and sediments
Name of your method:	A modified preparation method for TOC determination in suspended particulate samples
Name and reference of original method:	Hupach, S., 2009. Der neue TOC–Norm-Entwurf: Suspensionsmethode nach DIN EN 159,436. Analytik News, 1–3. https://analytik.news/fachartikel/pdf/shimadzu2.pdf
	Chua, A.M., Lee, Z.H., Tan, Q.A., 2022. Total Organic Carbon in Soil: A Comparison between Solid Sample Combustion and Suspension Methods. Application News 04-AD-0262-EN, SHIMADZU group https://www.shimadzu.com/an/sites/shimadzu.com.an/files/pim/pim_document_file/applications/application_note/14,574/an_04-ad-0262-en.pdf
Resource availability:	N/A

## Method details

### Rationale, materials, and analytical equipment

In many geoscientific studies, various techniques are used to directly determine and quantify carbon concentrations as total organic carbon (TOC), total inorganic carbon (TIC), and total carbon (TC). Carbon characterization and quantification are often essential parameters in studies that analyze soil and sediment samples obtained from varying environments. Such samples usually show a wide range of carbon concentrations and variable TOC to TIC ratios (e.g., [5,7,14,22,29,31,42]). Beyond geosciences, TOC quantification in particulate samples is of major concern for a broad field of environmental studies, including topics such as monitoring, management, and pollution with chemicals and plastics (e.g., [8,12,18,19,21,30]). In DIN EN 15,936, the European Committee for Standardization specifies TC, TIC, and TOC in particulate samples such as soils or sediments and describes two methods to quantify TOC [11]. According to this norm, TC refers to the amount of carbon present in a sample in the form of organic, inorganic, and elementary carbon, whereas TIC is designated as the amount of carbon released as CO_2_ by acid treatment, and TOC refers to the amount of carbon converted into CO_2_ by combustion which was not released as CO_2_ by preceding acid treatment. The two methods described in the norm for TOC determination are the *indirect procedure* and the *direct procedure*. The *indirect procedure* obtains TOC by subtracting the results of TIC measurements from those of TC measurements [11]. These complementary carbon fractions are often determined by CO_2_ quantification after dry combustion for TC contents and by the difference-on-ignition method followed by dry combustion or by acid treatment for TIC contents; detection and quantification of the evolved CO_2_ can be achieved by various techniques including non-dispersive infrared spectrometry, thermal conductivity, and conductivity change of NaOH solution (e.g., [3,5,6,17,25,26,34]). With the *direct procedure*, TOC is measured directly after carbonates have been removed from the samples by acid treatment [11]. Both procedures are commonly used in geoscientific studies (e.g., [27,43]), but pose inherent challenges. Applying the *indirect procedure* may lead to unreliable TOC results for samples with high TIC to TOC ratios, while the *direct procedure* will produce too low TOC values for samples that contain volatile organic substances [11]. Volatile organic substances such as polycyclic aromatic hydrocarbons (PAHs) evolve from the sample during acid treatment and sparging; evolved CO_2_ is quantified as purgeable organic carbon (POC). The non-purgeable organic carbon (NPOC) is the organic carbon that is not evolved from the sample during acid treatment and sparging [4,10,11]. Thus, for samples without a considerable content of hydrocarbons, NPOC is regarded as TOC [4].

Here we present a modified procedure to prepare sample suspensions for direct TOC measurements from soils and sediments. A modification of the preparation method proposed by the manufacturer [9,13] is needed, because quartz-rich samples are regularly analyzed and may cause problems with the dispersion tool that is used to homogenize the sample suspensions. We demonstrate the applicability and reliability of the modified method by quantification of TOC concentrations of five particulate certified reference materials (CRMs) and statistical assessment using descriptive and inferential statistics. A comparison shows that the modified method is more cost-efficient and environmentally friendly than the proposed preparation method, as it needs less sample material, saves hydrochloric acid and ultrapure water, and reduces waste. Beyond this, incomplete dissolution of dolomite, which may occur as a consequence of short acid treatment at low temperatures [23], is avoided by heating the acidified samples to 60 °C for one hour. Lastly, the application of the modified method is not limited to the Shimadzu device, but may also be useful for users that employ devices of other manufacturers to measure TOC in sample suspensions.

We used five particulate certified reference materials (CRMs) from different origins (Table 1) and with varying certified TOC contents (Table 2) to assess the applicability and reliability of the modified sample preparation procedure instead of performing a direct analytical comparison of the two methods. Therefore, we accept the certified concentrations, which were determined in interlaboratory round robin tests, as „true values” and demonstrate the reliability of our modified preparation procedure with statistical measures. We avoid a direct analytical comparison, because the preparation of samples that contain quartz grains using the proposed method caused the destruction of our dispersion tool.

**Table 1:** Official names, internal lab IDs, types of material, origin, and supplier of the used certified reference materials (see Table 2 for certified TOC concentrations).

Reference material	Lab ID	Type of material	Origin	Supplier/Reference
Sediment sample 23	RF23	Marine sediment	Tagus river estuary, Portugal	[35]
Sediment sample 31	RF31	Marine sediment	Harbor Zeebrugge, Belgium	[36]
SETOC sample 739	RF739	Fresh water sediment	Netherlands	[37]
ISE sample 955	RF955	Sandy soil	Mali	[38]
ISE sample 986	RF986	Sandy soil	Netherlands	[39]

**Table 2:** Certified TOC concentrations of the five CRMs (SD = standard deviation; MAD = median absolute deviation).

	Lab ID	n	Mean	SD	Median	MAD
TOC [mass-%]	RF23	51	1.600	0.195	1.620	0.128
	RF31	25	1.120	0.136	1.130	0.100
	RF739	71	4.060	0.638	4.010	0.428
	RF955	47	0.228	0.043	0.230	0.030
	RF986	126	1.770	0.211	1.760	0.147

In our lab, the CRMs were homogenized with a vibrating disc mill (Siebtechnik TS250) for 6 min to finely powder the sample material, oven-dried at 105 °C for > 4 h, and thereafter cooled down to room temperature in a desiccator to avoid moisture uptake during cooling. 15 aliquots of each CRM (5 with ∼ 25 mg, 5 with ∼ 50 mg, and 5 with ∼ 75 mg; see Supporting Information for full sample list with weights and C concentrations) were weighed into 40 ml glass vials using a semi-micro balance (VWR SM 2285Di-C) with a resolution of 0.01 mg.

TOC analyses were carried out with a Shimadzu TOC-L CPH analyzer and TOC contents were determined directly as NPOC. The system is equipped with an automatic sample charger that can host up to 68 individual 40 ml glass vials. The Shimadzu TOC-L CPH analyzer uses catalytic oxidation of sample suspensions at 680 °C in an O_2_ atmosphere and NDIR (non-dispersive infrared) detection of evolving CO_2_. Depending on the standard deviation (SD ≤ 1), 3–5 replicates of 90 µl were measured from each sample suspension using multiple injections to avoid bias caused by settling of particles. Repeated blank measurements of ultrapure water (*n* = 140) that are evenly included in our measurement routine regularly showed values of < 0.25 mg l ^−1^C. The resulting limit of blank (LOB = 0.3 mg l ^−1^C) describes the lowest carbon concentration that can be detected [1]. A multi-point calibration curve for a value range between 1 and 250 mg l ^−1^C was created by automatically diluting a 1000 mg l ^−1^C liquid standard (potassium hydrogen phthalate, C_8_H_5_KO_4_) to calibrate the 75 CRM measurements. The 1000 mg l ^−1^C liquid standard is additionally used to prepare control samples with concentrations of 5, 10, 30, 50, and 100 mg l ^−1^C that are routinely measured to monitor the proper functioning of the system.

### Originally proposed sample preparation procedure

**Fig. 1:**
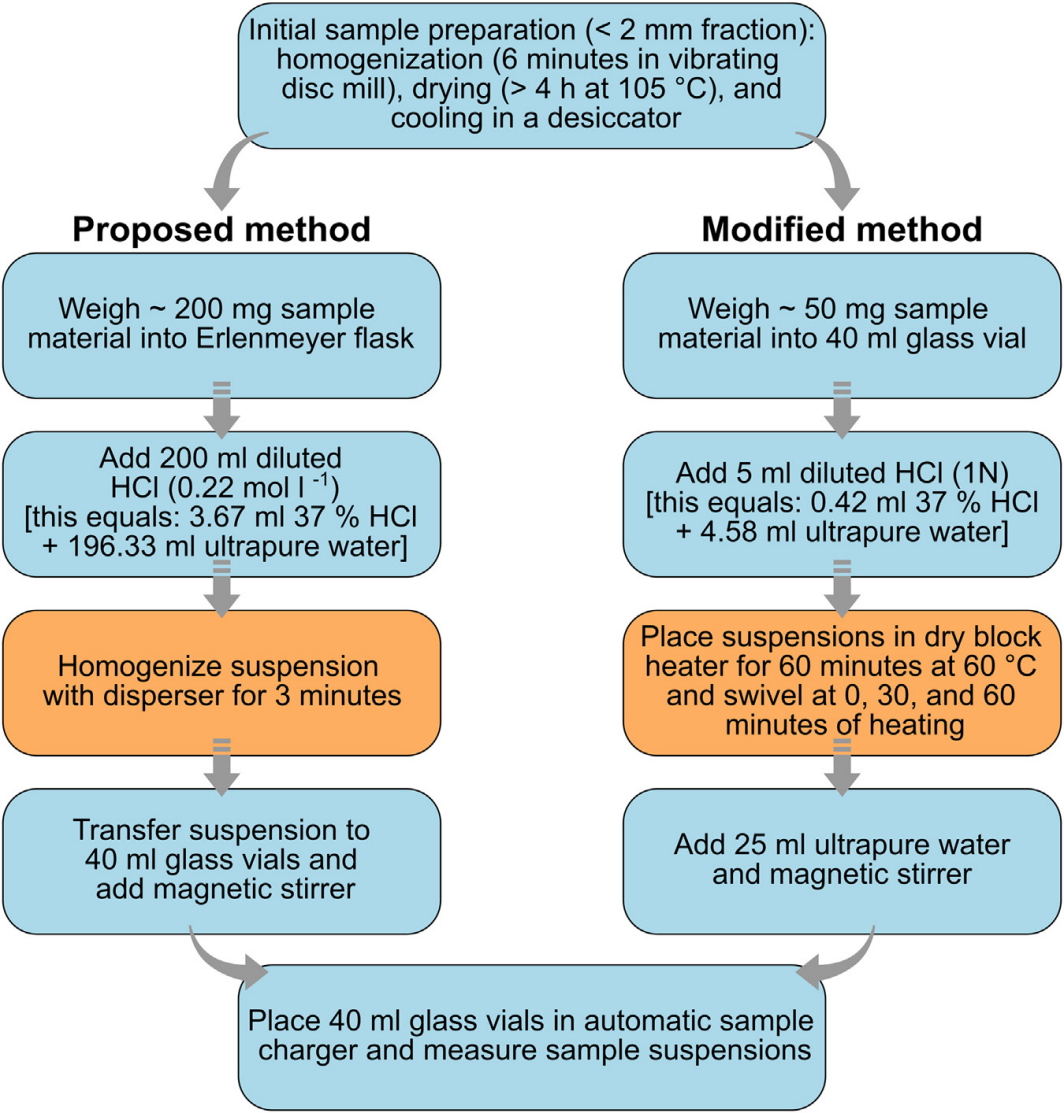
Flow chart showing the sample preparation steps of the proposed and the modified method; blue color denotes identical or nearly similar preparation steps and orange color indicates method-specific steps. A direct comparison demonstrates the savings potential of the modified preparation method: 0.42 ml concentrated HCl and 29.58 ml ultrapure water are needed to prepare one sample suspension with the modified method, while the proposed method requires 3.67 ml concentrated HCl and 196.33 ml ultrapure water. Except for swiveling at the beginning, after 30 min, and at the end of the acid treatment, the sample suspensions remain unattended in the dry block heater for 60 min (modified method). By contrast, the homogenization of each sample suspension for 3 min using a disperser (proposed method) requires active handling to change the suspensions every 3 min.

The sample preparation procedure proposed by the Shimadzu company (Fig. 1) suggests using ∼ 200 mg of sample material that is weighed into an Erlenmeyer flask and mixed with 200 ml diluted HCl (0.22 mol l ^−1^). Subsequently, the suspension is homogenized for three minutes at a speed of 17,000–18,000 rpm using a disperser, then transferred to 40 ml glass vials, and analyzed [9,13]. Additional steps such as heating of the acidified samples or a minimum reaction time for the acid treatment are not specified. A short duration of acid treatment at low temperatures, however, may lead to an incomplete dissolution of dolomite (CaMg[CO_3_]_2_), because dissolution rates of dolomite are more temperature controlled than those of CaCO_3_ [23].

### Modified sample preparation procedure

In contrast to the proposed sample preparation method, the modified method suggests to weigh the sample material directly into the 40 ml glass vials. Then, 5 ml 1 N HCl are added to the sample material to dissolve potentially present carbonates. The suspensions are swiveled and placed in an IKA dry block heater at 60 °C for one hour; swiveling is repeated after 30 and 60 min. Acid treatment of finely powdered material with 5 ml 1 N HCl at 60 °C for one hour guarantees the complete dissolution of calcium carbonate and dolomite for the chosen sample weights, as it contains ∼ 0.18 g concentrated HCl that has the capacity to dissolve ∼ 240 mg CaCO_3_ and ∼ 220 mg CaMg[CO_3_]_2_. After the dissolution of carbonates, the suspensions are mixed with 25 ml ultrapure water (≤ 0.055 µS cm^-1^) and magnetic stirrers are added to the glass vials to produce homogeneous suspensions during sample uptake.

The comparison of the proposed and the modified procedure for suspended sample preparation reveals that the modified method needs ca. one-quarter of the proposed sample material, a little more than a tenth of the amount of concentrated HCl to dissolute carbonates, and ca. 15% of the amount of ultrapure water (Fig. 1). Using this modification saved >242 ml concentrated HCl and 12.5 l ultrapure water for the 75 samples prepared for this study; consequently, also less waste was produced. Additionally, according to our experiences with the method proposed by the manufacturer, the use of a disperser for homogenizing sample material that contains a larger amount of quartz grains suspended in HCl may be problematic, because even finely powdered quartz grains caused the destruction of the dispersion tool of our disperser (IKA T 25 digital ULTRA-TURRAX).

## Method validation

**Fig. 2:**
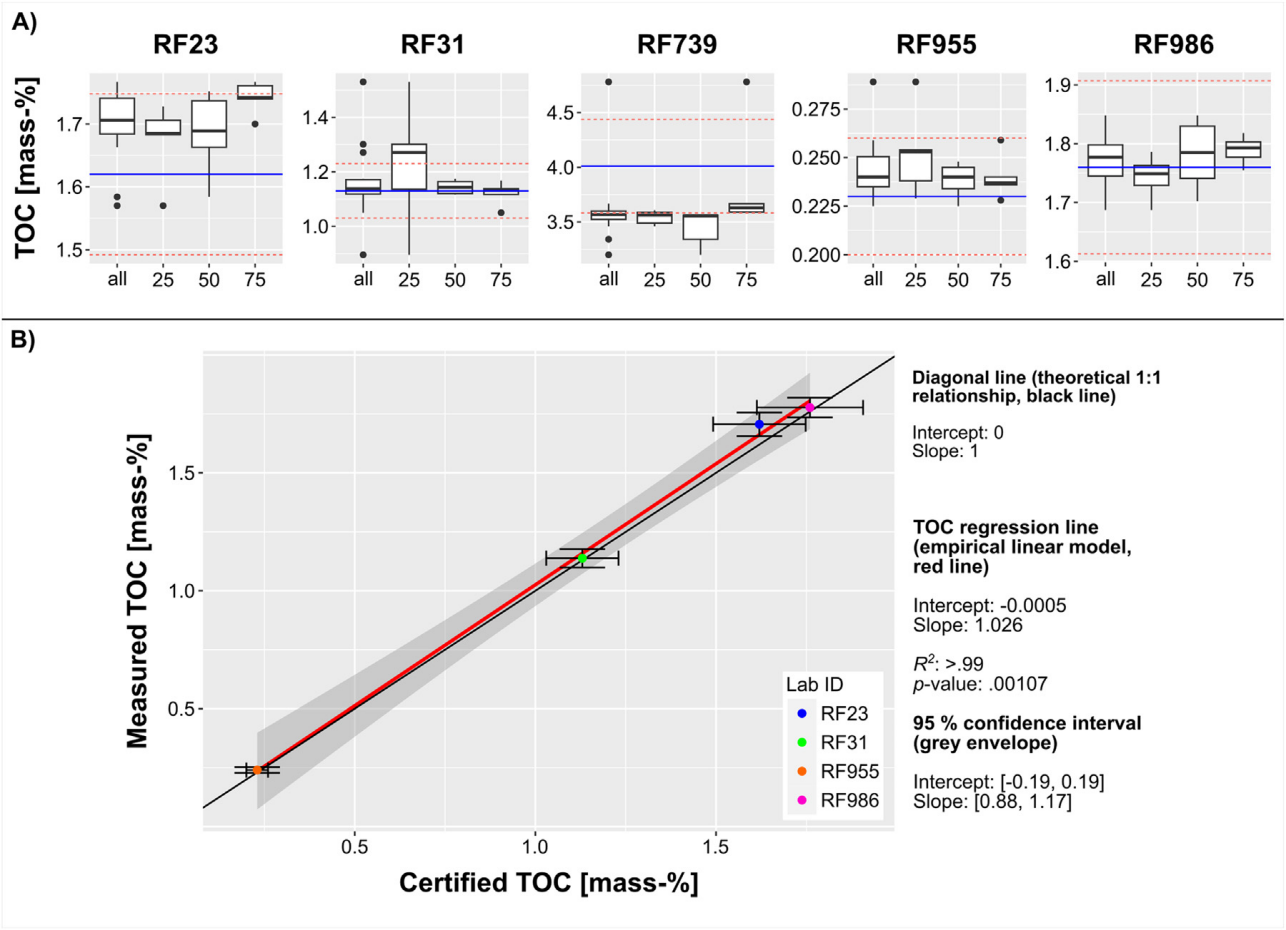
A) Box-and-whisker-plots in the style of Tukey [33] of measured TOC concentrations (rows of the layout). The blue lines mark the certified median values of the CRMs and the dashed red lines denote a scatter around the median of ± 1 certified MAD. Four box plots are presented for each CRM (columns) and labeled with the lab ID: the numbers on the x-axis refer to the five aliquots with weights of ∼ 25 mg, ∼ 50 mg, and ∼ 75 mg; “all” on the x-axis indicates that the box plot shows the data of the 15 aliquots of each CRM. The upper and lower hinges of the box represent the first and third quartiles (25th and 75th percentiles), the horizontal line in the box marks the median value, the whiskers represent 1.5 times the interquartile range, and the dots are outliers (cf. [24,33]). B) Linear regression analyses of measured and certified median TOC concentrations. Error bars refer to the scatter of ± 1 MAD around the measured and certified median values.

For comparison of the certified and measured concentrations, we focus on median values as a measure of central tendency and median absolute deviation (MAD) as a measure of statistical dispersion, because these are less affected by outliers than mean and standard deviation [20]. Due to the lack of raw data of the CRMs, we cannot directly compare the distributions of the certified and measured values. Therefore, for the purpose of this validation, we consider the median of the certified values and the scatter of ± 1 certified MAD as given target values for our measurements. To assess the consistency of the presented data over the full analytical range of the five CRMs, we used linear regression analyses of the determined and certified TOC concentrations in comparison with a diagonal line (intercept = 0; slope = 1). The slope of the diagonal line defines a 1:1 relationship between the response and the predictor variable; an increase of the predictor variable of 1 unit results in an increase of 1 unit of the response variable. We interpret the difference between the slope of the regression line (empirical linear model) and those of the diagonal line (theoretical 1:1 relationship) to be statistically significant if 1, i.e. the slope of the diagonal line, is not within the 95% confidence interval of the empirical model (cf. Fig. 2B). To control the quality of their measured data, Simandl et al. [32] apply percent difference (%diff) between measured and certified concentrations of chemical elements as a measure of accuracy, and relative standard deviation (%RSD) of repeated measurements as a measure of precision. Accuracy, or in our case percent bias, determines the relative proximity of the measured values to the certified values of the CRMs. Precision, i.e. repeatability, determines the degree to which repeated measurements, obtained with the same device and under identical conditions, yield the same results. Simandl et al. [32] use the measured and certified mean values to calculate accuracy, and the mean values and standard deviations of the measurements to calculate precision. We adopt this approach to further evaluate the quality of the TOC measurements, but use the measured and certified median values to calculate accuracy, and the median and MADs of the measured values to calculate precision. Consequently, in this case, accuracy is determined by the deviation from the certified median values, expressed as % bias (Eq. (1); x̃_meas._ = median of the measured values; x̃_cert._ = certified median), while precision, expressed as % variability, represents the deviation within 1 MAD of the median (Eq. (2); MAD_meas._ = median absolute deviation of the measured values).

(1) $$\%bias=\left(\frac{\left|({\tilde{x}}_{meas.\;}-\;{\tilde{x}}_{cert.})\right|}{{\tilde{x}}_{cert.}}\right)*100$$

(2) $$\%variability=\left(\frac{MAD_{meas.}}{{\tilde{x}}_{meas.\;}}\right)*100$$

Data analyses and plotting of graphs were undertaken in R [28] using dplyr [41], ggplot2 [40], ggpubr [16], and gridExtra [2]. Single plots were grouped in Inkscape 1.2.1 [15].

The median values of the 15 aliquots per CRM of the measured TOC concentrations (summarized in Table 3; complete data in Supporting Information) are usually close to the certified median values (Table 2) and, with the exception of the median TOC content of RF739, all median values of the 15 aliquots per measured CRM fall into the scatter of the certified median ± 1 certified MAD (Fig. 2A). The analyses of three different sample weights (∼ 25 mg, ∼ 50 mg, and ∼ 75 mg) per CRM allow assessment of the influence of the sample weight on the data quality of the TOC analyses. The comparison shows that, except for RF739, reliable results are obtained with ∼ 50 mg sample weight. A higher sample weight of ∼ 75 mg has no substantial positive impact on the data quality (Fig. 2A). In terms of absolute TOC concentrations, the values of the sample suspensions ranged between ∼ 1.8 mg l ^−1^ (∼ 22 mg of RF955) and ∼ 96 mg l ^−1^ (∼ 79 mg of RF739; with one outlier with ∼ 120 mg l ^−1^), which is at least six times higher than the determined LOB (limit of blank) of 0.3 mg l ^−1^C (*n* = 140).

**Table 3:** Measured TOC concentrations of the five CRMs (SD = standard deviation; MAD = median absolute deviation).

	Lab ID	n	Mean	SD	Median	MAD
TOC [mass-%]	RF23	15	1.700	0.059	1.706	0.050
	RF31	15	1.164	0.136	1.138	0.039
	RF739	15	3.613	0.345	3.568	0.058
	RF955	15	0.244	0.016	0.240	0.012
	RF986	15	1.771	0.045	1.777	0.042

The essential underestimation of the median TOC content of RF739 (Fig. 2A; Table 3) is due to the presence of polycyclic aromatic hydrocarbons (PAHs) in the sample material [37]. These volatile organic substances evolve as POC from the sample during acid treatment [11] and even the homogenizing process may release these compounds [4].

A linear regression analysis of measured and certified median TOC concentrations compared to a diagonal line allows evaluation of the consistency of the data over the analytical range. The proximity of the diagonal line (theoretical 1:1 relationship) and the empirical linear model of the TOC concentrations generally demonstrates the high level of agreement for the analytical range (Fig. 2B). This is further supported by the error bars, i.e. median values ± 1 MAD, which are wider than the distance between the lines and also often fully cross them, demonstrating that the variation of the 15 aliquots per CRM measurement is usually larger than the deviation of the two lines. Due to the presence of PAHs, the values of RF739 were excluded from regression analysis. Therefore, the regression line of the TOC covers the range of the analyzed concentrations between ∼ 0.2 and ∼ 1.8 mass-% C. The intercept of the regression line lies near 0 and its slope is slightly above 1, indicating a minor tendency to overestimate the TOC content with increasing absolute C concentrations (Fig. 2B). However, the observed deviation of the slope of the linear model and the diagonal line are not statistically significant, because the 95% confidence interval of the linear model includes 1, i.e., the slope of the diagonal line (Fig. 2B).

The TOC measurements yielded accurate (%bias between 0.7 and 5.3)—except for RF739 that contains PAHs (11.0%bias)—and precise (%variability between 1.6 and 4.9) results (Table 4). Accordingly, between 94.7 and 99.3% of the certified median values were retrieved by the repeated TOC measurements of the four CRMs that contain negligible amounts of PAHs. The precision shows that the measurements scatter between ± 1.6 and ± 4.9% around the respective median values.

**Table 4:** Precision and Accuracy of the measured TOC.

	Lab ID	n	Accuracy [%bias]	Precision [%variability]
TOC	RF23	15	5.3	3.0
	RF31	15	0.7	3.4
	RF739	15	11.0	1.6
	RF955	15	4.3	4.9
	RF986	15	1.0	2.3

## Conclusions

The applicability and reliability of a modified sample preparation method for direct TOC determinations of suspended particulate samples using a Shimadzu TOC-L CPH system is demonstrated by a combination of TOC measurements of five certified reference materials (CRMs) with varying TOC concentrations and statistical measures. The results show a high degree of agreement between the measured and the certified median values. With the exception of one CRM that contains polycyclic aromatic hydrocarbons (PAHs), the median values of all analyzed aliquots of the CRMs that contain negligible amounts of volatile organic substances lie within the scatter of ± 1 MAD around the certified median. This is also confirmed by a high precision and accuracy for the TOC analyses of these CRMs. Even comparable smaller sample weights of ∼ 50 mg of materials that contain only minor organic carbon amounts produce accurate and precise TOC results. Based on these findings, we recommend applying the modified procedure to prepare suspended particulate samples for the direct measurement of TOC concentrations with a Shimadzu TOC-L CPH analyzer or with comparable devices from other manufacturers. The modified sample preparation procedure requires less sample material, saves acid and ultrapure water, reduces waste, and avoids incomplete dissolution of dolomite by heating the acidified samples. Beyond this, it avoids the risk of destruction of the dispersion tool, as may occur during the preparation of quartz-rich samples suspended in HCl.

## Ethics statements

The authors declare that no human participants, human data or human tissue are involved in the study.

## CRediT author statement

**M.N.:** Conceptualization, Formal analysis, Validation, Visualization, Writing – original draft, Writing – review & editing. **F.B.:** Formal analysis, Validation, Visualization, Writing – review & editing. **P.H.:** Conceptualization, Formal analysis, Validation, Writing – review & editing.

## Declaration of competing interest

The authors declare that they have no known competing financial interests or personal relationships that could have appeared to influence the work reported in this paper.

## Data Availability

Data is provided in the Supporting Information of this article

## Appendix Supplementary materials

Image, application 1
